# Mitochondrial COI and 16S rDNA barcoding improve species delimitation of ixodid ticks in Peninsular Malaysia

**DOI:** 10.1186/s13071-026-07546-3

**Published:** 2026-07-03

**Authors:** Nurul Aini Husin, Van Lun Low, Muhammad Haiqal Syarriman AbdulRahim, Muhammad Rasul Abdullah Halim, Auni Atikah AbdulHalim, Muhammad Al Amin Mohd-Redzuan, Edley A. Jiliun, Ahmad Khusaini Mohd Kharip Shah, Benjamin L. Makepeace, Zubaidah Ya’cob

**Affiliations:** 1 Higher Institution Centre of Excellence, Tropical Infectious Diseases Research & Education Centre (TIDREC), Universiti Malaya, 50603 Kuala Lumpur, Malaysia; 2 Institute for Advanced Studies, Universiti Malaya, 50603 Kuala Lumpur, Malaysia; 3 Institute of Biological Sciences, Faculty of Science, Universiti Malaya, 50603 Kuala Lumpur, Malaysia; 4 Department of Wildlife and National Parks, Peninsular Malaysia, KM10, Jalan Cheras, 56100 Kuala Lumpur, Malaysia; 5 Institute of Infection, Veterinary and Ecological Sciences, University of Liverpool, Liverpool, L3 5RF UK

**Keywords:** Hard ticks, Ixodidae, DNA barcoding, COI, 16S rDNA, Species delimitation, Vector surveillance

## Abstract

**Background:**

Hard ticks (Acari: Ixodidae) are major vectors of zoonotic pathogens affecting humans and animals. Accurate species identification is essential for surveillance and disease risk assessment but is often hindered by morphological similarity and intraspecific variation. In Malaysia, tick studies have largely relied on morphology or single-gene barcoding, with limited taxonomic and geographic coverage.

**Methods:**

This study applied a multilocus mitochondrial approach using cytochrome c oxidase I (COI) and 16S ribosomal DNA (rDNA) to evaluate species boundaries among ixodid ticks from Peninsular Malaysia. Ticks were collected from wild and domestic hosts between 2022 and 2023. A total of 319 specimens representing 14 morphologically defined species were analysed using COI and 16S rDNA sequencing, with additional reference sequences retrieved from GenBank for comparative analyses. Phylogenetic reconstruction, barcode gap assessment, and four species delimitation methods (ASAP, ABGD, bPTP, and GMYC) were employed to assess genetic divergence and identification accuracy.

**Results:**

Molecular analyses were largely congruent with morphological identification. Distance-based delimitation methods (ASAP and ABGD) recovered operational taxonomic units (OTUs) largely consistent with morphologically defined species, whereas tree-based approaches (bPTP and GMYC) inferred substantially higher numbers of OTUs, reflecting sensitivity to intraspecific mitochondrial structuring. These additional subdivisions are interpreted conservatively as population-level genetic differentiation rather than evidence of distinct species. Clear barcode gaps were observed for COI and the concatenated COI + 16S rDNA datasets, whereas partial overlap occurred with 16S rDNA alone. COI demonstrated the highest and most consistent performance for routine species identification, while concatenated datasets improved phylogenetic resolution but reduced assignment clarity under strict barcoding criteria.

**Conclusions:**

Overall, COI remains the most effective mitochondrial marker for routine species identification of ixodid ticks in Peninsular Malaysia. These findings highlight the value of integrative approaches combining morphology and molecular data to strengthen tick taxonomy and support surveillance of tick-borne pathogens in the region.

**Graphical Abstract:**

**Figure:**
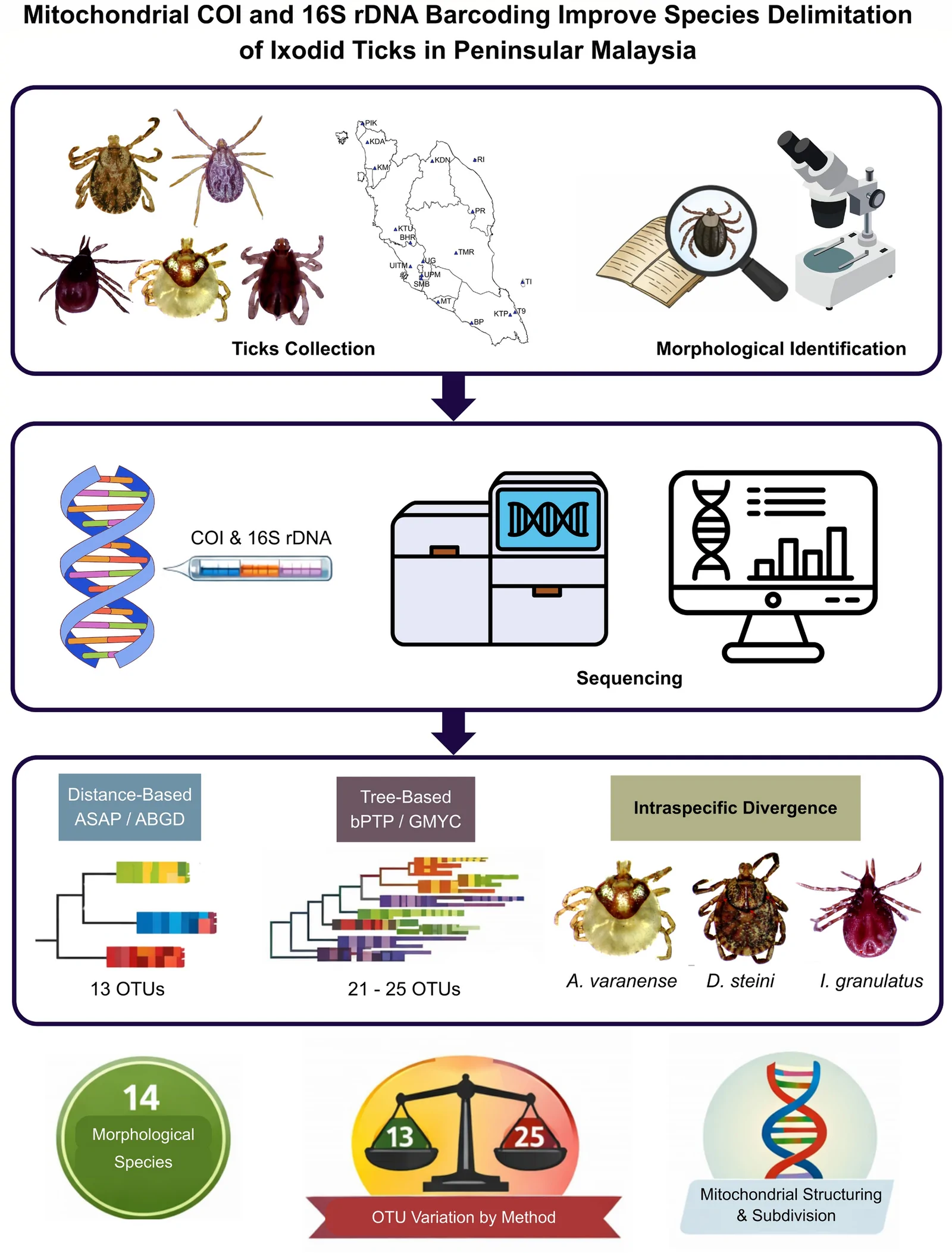

**Supplementary Information:**

The online version contains supplementary material available at https://doi.org/10.1186/s13071-026-07546-3.

## Background

Hard ticks are obligate blood-feeding ectoparasites of vertebrates and important vectors of diverse pathogens, including bacteria, viruses, and protozoa [1]. Accurate species identification is essential for understanding their role in disease transmission, developing effective control strategies, and assessing their ecological impact. Tick-borne diseases, such as Lyme disease, babesiosis, rickettsiosis, and tick-borne encephalitis, continue to pose significant public health and veterinary concern worldwide [2], underscoring the need for reliable taxonomic resolution. Traditionally, tick species identification has depended on morphological characters, such as mouthpart structure, scutal ornamentation, and coxal spurs [3]. Although these diagnostic traits are informative, morphological variation driven by intraspecific polymorphism, phenotypic plasticity, or ontogenetic changes can impede accurate identification [2, 4]. In addition, close morphological similarities and fine-scale genetic structuring among certain tick taxa further complicate precise species delimitation [5].

Molecular techniques, especially DNA barcoding, have transformed tick taxonomy by enabling more precise species identification and better understanding of phylogenetics [6]. Standardized mitochondrial markers, especially COI and 16S rDNA, are widely used for species discrimination in Ixodidae [7, 8]. As in numerous other metazoans, COI has been extensively applied as a universal barcode for distinguishing species across major tick genera, including *Rhipicephalus*, *Haemaphysalis*, *Amblyomma*, *Dermacentor*, and *Ixodes* [7]. Moreover, 16S rDNA has proven important in phylogenetic analyses because of its conserved sequence and capacity to clarify relationships within Ixodidae [9]. However, reliance on a single mitochondrial marker may underestimate patterns of genetic variation or obscure fine-scale genetic structuring, highlighting the potential value of multilocus approaches [10].

In Malaysia, tick research has largely focused on tick surveillance, species identification, and molecular screening of tick-borne pathogens [11, 12]. Although molecular approaches have increasingly been applied in recent years, particularly for pathogen screening and species confirmation, comprehensive molecular-based taxonomic and integrative systematic studies of ticks remain comparatively underrepresented [13]. The country hosts diverse tick fauna, including medically and veterinary important genera such as *Rhipicephalus*, *Haemaphysalis*, *Amblyomma*, and *Ixodes*, many of which are linked to wildlife, domestic animals, and humans [14]. However, earlier research has primarily depended on morphological methods, which may lead to misidentification in morphologically similar taxa. The use of DNA barcoding in Malaysian tick research remains limited. While COI has been employed in some studies for species discrimination [13, 15], the potential of 16S rDNA as a complementary marker remains underexplored. This limits the resolution of molecular taxonomy, particularly for detecting fine-scale genetic structuring within species.

Therefore, this study applied an integrative taxonomic approach combining morphological identification with mitochondrial DNA barcoding (COI and 16S rDNA) to evaluate species boundaries, genetic divergence, and phylogenetic relationships of ixodid ticks collected from diverse hosts and habitats in Peninsular Malaysia. We specifically compared the performance of individual and concatenated markers for accurate species delimitation and detection of fine-scale genetic structuring across five tick genera. By establishing a multilocus mitochondrial reference framework, this study aims to strengthen baseline tick taxonomy and support molecular surveillance efforts for vector identification and monitoring of tick-borne pathogens in the region.

## Methods

### Sample collection and morphological identification

**Fig. 1:**
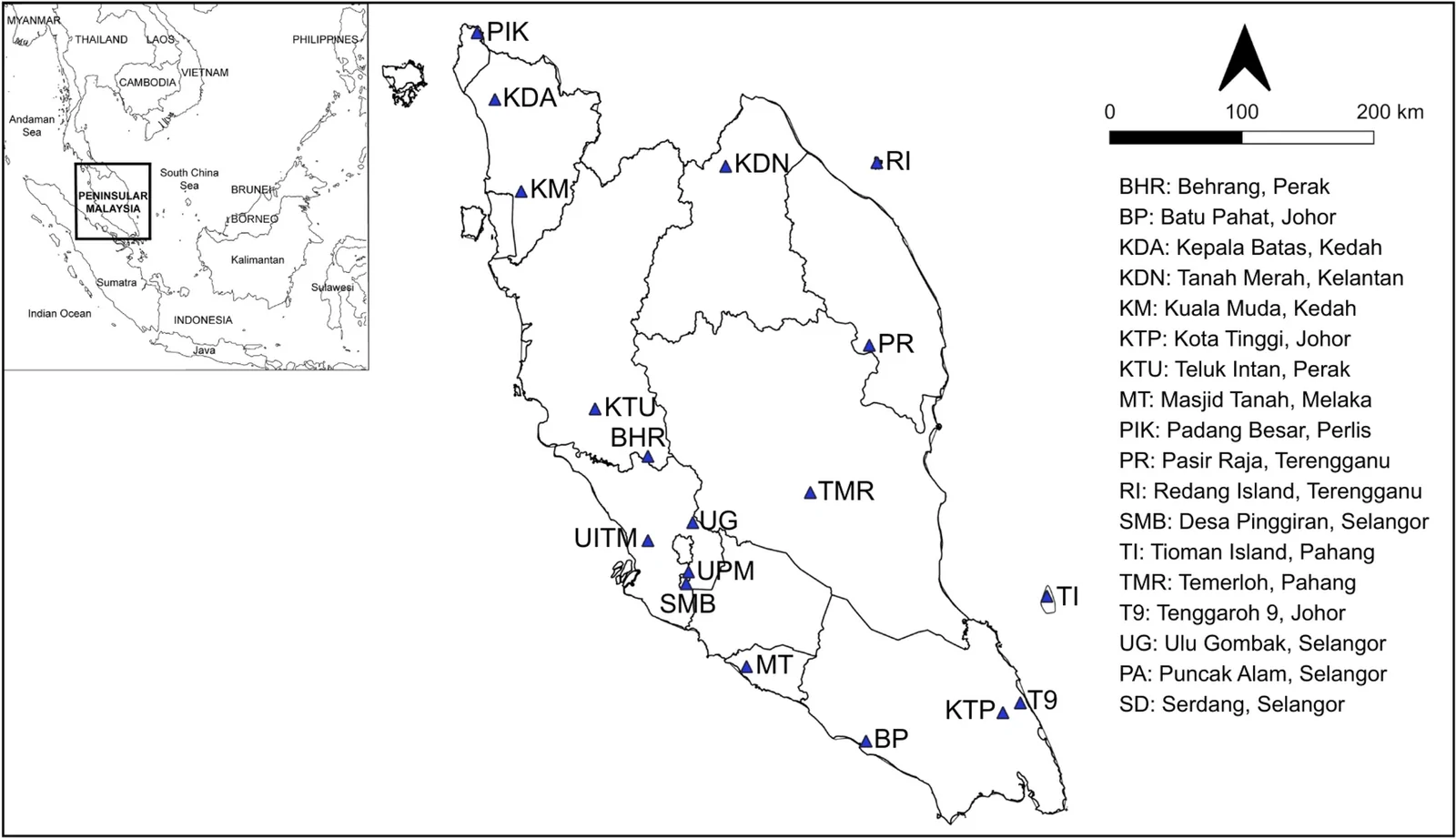
Geographic distribution of the 18 sampling sites across Peninsular Malaysia where tick specimens were collected between 2022 and 2023

The experimental design and procedures of this study were approved by the Universiti Malaya Institutional Biosafety and Biosecurity Committee (Approval codes: UMIBBC/PA/R/TNCPNI /TIDREC-004/2020 and UMIBBC/NOI/R/TNCPNI/TIDREC-007/2024). Tick specimens were collected between 2022 and 2023 from 18 sampling sites [16] across Peninsular Malaysia (Fig. 1). Ticks were carefully removed from animal hosts using fine-tipped forceps and placed into appropriately sized sterile plastic tubes. Non-engorged ticks were stored individually in 2 mL cryovials, while engorged specimens were preserved in universal tubes containing 70% ethanol. Each sample was labeled with a unique host identification code, collection date, and sampling location to ensure proper traceability. Ticks at all life stages, including larvae, nymphs, and adults, were examined under stereomicroscopes and compound microscopes for detailed morphological assessment. Species identification was carried out using established taxonomic keys [17–19].

### DNA extraction

Prior to extraction, ticks preserved in ethanol were rinsed with sterile Milli-Q water to remove residual ethanol and air-dried briefly. Genomic DNA was extracted from each tick specimen using the DNeasy Blood & Tissue Kit (Qiagen, Germany) for adults and the QIAamp DNA Micro Kit (Qiagen, Germany) for nymphs and larvae, following the manufacturers’ protocols. Each specimen was homogenized in 180 µL lysis buffer with 20 µL Proteinase K and incubated overnight at 56 °C. The following steps were carried out following the manufacturer’s instructions. DNA was eluted in 50 µL of elution buffer and stored at  −80 °C until further use. DNA concentration and purity were measured using a Qubit dsDNA HS Assay Kit and a NanoDrop 2000 spectrophotometer (Thermo Fisher Scientific, United States).

### PCR and DNA sequencing

Two mitochondrial markers, COI and 16S rDNA, were amplified from genomic DNA using published primer sets (Table 1). PCR reactions were performed in 25 µL volumes using DreamTaq Green Master Mix (Thermo Fisher Scientific, USA) according to the manufacturer’s protocol. Nuclease-free water was included as a negative control in each PCR run. Amplifications were conducted in a SimpliAmp^™^ Thermal Cycler (Applied Biosystems, USA). PCR products were visualized on 1% agarose gels stained with GelRed (GeneAll Biotechnology, Korea). Amplicons of the expected size were purified and sequenced bidirectionally by Apical Scientific Sdn. Bhd. (Selangor, Malaysia) using the same primer pairs.

**Table 1:** Details of mitochondrial gene amplification, including primer pairs, nucleotide sequences (5′–3′), expected amplicon sizes, PCR cycling conditions, and original references for COI and 16S rDNA markers

Marker	Primer	Sequence (5′–3′)	Amplicon size (bp)	PCR cycling conditions	References
COI*	cox1F/cox1R (first reaction)	GGAACAATATATTTAATTTTTGG/ATCTATCCCTACTGTAAATATATG	732	95 °C 5 min; 40 × (95 °C 30 s, 55 °C 1 min, 72 °C 1 min); 72 °C 10 min	[40]
	C1-J-1718/C1-N-2329 (second reaction)	GGAGGATTTGGAAATTGATTAGTTCC/ACTGTAAATATATGATGAGCTCA	612		[44]
16S rDNA	16S + 1/16S-1	CTGCTCAATGAATATTTAAATTGC/CGGTCTAAACTCAGATCATGTAGG	460	94 °C 2 min; 10 × (92 °C 1 min, 48 °C 1 min, 72 °C 1 min); 32 × (94 °C 1 min, 54 °C 1 min, 72 °C 1 min); 72 °C 7 min	[9]

^*^Nested PCR amplification strategy adapted from Low et al. [15], who demonstrated improved PCR amplification performance in tick samples

### Phylogenetic analyses

A total of 754 sequences were included in the analyses. Of these, 638 sequences were generated in this study, comprising 319 COI and 319 16S rDNA sequences. The newly generated sequences have been submitted to GenBank under accession numbers PZ509685–PZ510004 (COI) and PZ499456–PZ499775 (16S rDNA). Additionally, 116 reference sequences from various species retrieved from GenBank were included for comparison (Table 2). All reference sequences were selected based on Basic Local Alignment Search Tool (BLAST) analysis from GenBank and morphological identification. *Argas persicus* (Oken, 1818) was used as the outgroup taxon.

**Table 2:** Summary of tick specimens analysed in this study, including sampling location, life stage, host species, and total number of sequences per species

Tick species	Location	Life stage	Host species	Total sequence (*n* = 319)
*Amblyomma cordiferum* (Neumann, 1899)	Batu Pahat, Johor (BP)	Af	*Malayopython reticulatus*	4
		Af	*Ophiophagus bungarus*	3
	Redang Island, Terengganu (RI)	L	*Maxomys rajah*	1
		N	*Rattus tiomanicus*	5
		L	*Rattus tiomanicus*	8
		L	*Tupaia glis*	4
	Tanah Merah, Kelantan (KDN)	Af	*Malayopython reticulatus*	1
	Tioman Island, Pahang (TI)	N	*Leopoldamys vociferans*	2
		L	*Leopoldamys vociferans*	1
		N	*Maxomys surifer*	2
		L	*Maxomys surifer*	3
		N	*Rattus tiomanicus*	1
	Kota Tinggi, Johor (KTP)	L	*Rattus tanezumi*	2
*Amblyomma helvolum* (Koch, 1844)	Batu Pahat, Johor (BP)	Am	*Ophiophagus bungarus*	1
*Amblyomma varanense* (Supino, 1897)	Batu Pahat, Johor (BP)	Am	*Ophiophagus bungarus*	1
		Af	*Ophiophagus bungarus*	2
	Redang Island, Terengganu (RI)	Am	*Varanus salvator*	1
		Af	*Varanus salvator*	1
	Tanah Merah, Kelantan (KDN)	N	*Malayopython reticulatus*	4
		Am	*Malayopython reticulatus*	1
*Dermacentor auratus* (Supino, 1897)	Kepala Batas, Kedah (KDA)	Am	*Sus scrofa*	3
		Af	*Sus scrofa*	2
	Padang Besar, Perlis (PIK)	Am	*Sus scrofa*	2
		Af	*Sus scrofa*	2
	Kota Tinggi, Johor (KTP)	Am	*Sus barbatus*	1
		Af	*Sus barbatus*	2
*Dermacentor steini* (Schulze, 1933)	Behrang, Perak (BHR)	Am	*Gonocephalus bellii*	1
	Masjid Tanah, Melaka (MT)	Am	*Sus scrofa*	2
	Puncak Alam, Selangor (PA)	Am	*Sus scrofa*	1
*Haemaphysalis bispinosa* (Neumann, 1897)	Kepala Batas, Kedah (KDA)	L	*Macaca fascicularis*	1
		Af	*Sus scrofa*	1
*Haemaphysalis shimoga* (Trapido & Hoogstraal, 1964)	Pasir Raja, Terengganu (PR)	Af	*Leopoldamys vociferans*	2
		L	*Leopoldamys vociferans*	1
		N	*Lophura erythrophthalma*	1
		L	*Lophura erythrophthalma*	2
		N	*Niviventer cremoriventer*	3
		N	*Sundamys muelleri*	1
*Haemaphysalis hystricis* (Supino, 1897)	Behrang, Perak (BHR)	N	*Copsychus malabaricus*	1
		N	*Maxomys whiteheadi*	1
	Padang Besar, Perlis (PIK)	Am	*Sus scrofa*	5
		Af	*Sus scrofa*	1
	Pasir Raja, Terengganu (PR)	L	*Gallus gallus*	1
		N	*Leopoldamys vociferans*	1
		N	*Lophura erythrophthalma*	1
		L	*Lophura erythrophthalma*	3
		N	*Maxomys rajah*	1
		N	*Maxomys surifer*	1
		L	*Polyplectron malacense*	2
	Ulu Gombak, Selangor (UG)	Af	*Canis familiaris*	1
*Haemaphysalis nadchatrami* (Hoogstraal, Trapido and Kohls, 1965)	Batu Pahat, Johor (BP)	Am	*Sus scrofa*	2
		Af	*Sus scrofa*	5
	Kepala Batas, Kedah (KDA)	Am	*Sus scrofa*	3
		Af	*Sus scrofa*	4
	Masjid Tanah, Melaka (MT)	Am	*Sus scrofa*	4
		Af	*Sus scrofa*	4
	Kota Tinggi, Johor (KTP)	Am	*Sus barbatus*	5
		Af	*Sus barbatus*	6
		N	*Sus barbatus*	1
	Serdang, Selangor (SD)	Am	*Sus scrofa*	1
*Haemaphysalis semermis* (Neumann, 1940)	Behrang, Perak (BHR)	N	*Pellorneum malaccense*	1
	Pasir Raja, Terengganu (PR)	N	*Gallus gallus*	3
		L	*Gallus gallus*	4
		N	*Leopoldamys vociferans*	2
		L	*Leopoldamys vociferans*	2
		N	*Lophura erythrophthalma*	3
		L	*Lophura erythrophthalma*	2
		L	*Lophura rufa*	3
		N	*Maxomys rajah*	3
		N	*Maxomys surifer*	5
		N	*Niviventer cremoriventer*	1
		L	*Niviventer cremoriventer*	2
		N	*Polyplectron malacense*	5
		L	*Polyplectron malacense*	7
		N	*Tragulus javanicus*	1
		N	*Tupaia glis*	4
*Haemaphysalis wellingtoni* (Nuttall and Warburton, 1908)	Desa Pinggiran, Selangor (SMB)	L	*Macaca fascicularis*	4
	Tanah Merah, Kelantan (KDN)	N	*Gallus gallus domesticus*	5
		Am	*Gallus gallus domesticus*	5
		Af	*Gallus gallus domesticus*	5
		L	*Gallus gallus domesticus*	5
	Teluk Intan, Perak (KTU)	Am	*Gallus gallus domesticus*	4
		Af	*Gallus gallus domesticus*	3
		N	*Gallus gallus domesticus*	2
		L	*Gallus gallus domesticus*	2
	Temerloh, Pahang (TMR)	Am	*Gallus gallus domesticus*	4
		Af	*Gallus gallus domesticus*	2
		N	*Gallus gallus domesticus*	2
		L	*Gallus gallus domesticus*	4
	Kota Tinggi, Johor (KTP)	Am	*Gallus gallus*	5
		Af	*Gallus gallus*	5
		N	*Gallus gallus*	6
		L	*Gallus gallus domesticus*	1
*Haemaphysalis* sp.	Pasir Raja, Terengganu (PR)	L	*Tragulus javanicus*	8
		N	*Tupaia glis*	1
	Tioman Island, Pahang (TI)	N	*Tupaia glis*	1
*Ixodes granulatus* (Supino, 1897)	Pasir Raja, Terengganu (PR)	Af	*Leopoldamys vociferans*	4
		Af	*Maxomys surifer*	1
		Af	*Sundamys muelleri*	1
		N	*Sundamys muelleri*	1
	Redang Island, Terengganu (RI)	N	*Crocidura malayana*	2
		Am	*Maxomys rajah*	2
		Af	*Maxomys rajah*	2
		N	*Maxomys rajah*	1
		Af	*Rattus tiomanicus*	4
		N	*Rattus tiomanicus*	3
		Am	*Tupaia glis*	2
		Af	*Tupaia glis*	6
		N	*Tupaia glis*	2
	Tenggaroh 9, Johor (T9)	Af	*Rattus tanezumi*	3
		N	*Rattus tanezumi*	1
		Af	*Tupaia glis*	1
	Tioman Island, Pahang (TI)	Af	*Lariscus insignis*	1
		Am	*Leopoldamys vociferans*	7
		Af	*Leopoldamys vociferans*	5
		N	*Leopoldamys vociferans*	2
		Am	*Maxomys surifer*	3
		Af	*Maxomys surifer*	4
	Ulu Gombak, Selangor (UG)	Af	*Sundamys muelleri*	4
*Rhipicephalus microplus* (Canestrini, 1888)	Kuala Muda, Kedah (KM)	Am	*Bos indicus*	4
		Af	*Bos indicus*	1

Life stage abbreviations: *Af* adult female, *Am* adult male, *N* nymph, *L* larva

Sequences were assembled and aligned using ClustalW [20] and edited in BioEdit v7.2.5 [21]. The aligned datasets comprised 620 bp for COI and 460 bp for 16S rDNA. Concatenation of COI and 16S rDNA sequences was performed using Molecular Evolutionary Genetics Analysis (MEGA) software v11.0.11. The optimal nucleotide substitution model for the concatenated dataset was determined using Model Selection in MEGA v11.0.11 based on the Bayesian Information Criterion (BIC). The Tamura–Nei model with gamma-distributed rate variation (TN93 + G) was selected for subsequent analyses.

Phylogenetic trees were reconstructed for individual gene datasets (COI and 16S rDNA) and the concatenated datasets. Maximum Likelihood (ML) analysis was conducted using RAxML-HPC BlackBox on the Cyber Infrastructure for Phylogenetic Research (CIPRES) Science Gateway v3.3, with automated model selection using BIC criterion [22]. Bayesian Inference (BI) analysis was performed using Bayesian evolutionary analysis by sampling trees (BEAST) software v10.5.0 [23]. The aligned sequence files in FASTA format were converted into Nexus files using BEAuti software v10.5.0, and annotated with the Hasegawa–Kishono–Yano (HKY) substitution model, with a gamma distribution rate of 4 and a speciation yule process prior with strict clock. The BEAST analysis used Markov Chain Monte Carlo (MCMC) for 10 million iterations with subsampling every 100 iterations. The maximum clade credibility (MCC) tree was generated using TreeAnnotator v10.5.0 with a burn-in rate of 25%. All trees were visualized in FigTree v1.4.4 and edited in the Interactive Tree of Life (iTOL) [24] and Adobe Illustrator 2020.

### Pairwise genetic distance analysis

The COI, 16S rDNA, and concatenated sequences were aligned by species based on the phylogenetic analysis. Uncorrected pairwise distances and Kimura 2-parameter (K2P) intra- and interspecific distances were calculated using MEGA v11.0.11 [25] and TaxonDNA Species Identifier software v1.7.7 [26]. Barcoding gaps were assessed by comparing intra- and interspecific genetic distances. In the first approach, a species boundary was inferred when the minimum interspecific distance exceeded the maximum intraspecific distance. Pairwise distances were calculated from sequences with at least 300 bp overlap, including all intra- and interspecific comparisons. To reduce the influence of outliers, 5% of the largest intraspecific distances and 5% of the smallest interspecific distances were excluded to calculate the 90% pairwise genetic distances [26, 27]. In the second approach, the magnitude of the barcoding gap was analyzed by plotting the farthest conspecific distance versus nearest neighbor (NN) and the mean conspecific distance versus NN [28].

### Species similarity analysis

Three similarity-based DNA distance approaches, Best Match (BM), Best Close Match (BCM) and All Species Barcode (ASB) [26], were conducted using TaxonDNA Species Identifier software v1.7.7 based on the Kimura 2-parameter model. Reference sequences retrieved from GenBank (Table 2) were used for comparison with the query sequences. These analyses were performed separately for COI, 16S rDNA, and the concatenated dataset to compare marker performance.

### Species delimitation analysis

Species delimitation was conducted using four complementary approaches, comprising two distance-based methods and two tree-based coalescent models. Distance-based analyses included Assemble Species by Automatic Partitioning (ASAP) [29] and Automatic Barcode Gap Discovery (ABGD) [30]. ASAP was performed using the online server (https://bioinfo.mnhn.fr/abi/public/asap/) under default parameters. ABGD analyses were conducted via the web interface (https://bioinfo.mnhn.fr/abi/public/abgd/) using the Kimura 2-parameter (K2P) distance model and default prior settings. Both methods infer species boundaries by detecting barcode gaps in the distribution of pairwise genetic distances.

Tree-based delimitation analyses were performed using Bayesian Poisson Tree Processes (bPTP) [31] and the General Mixed Yule-Coalescent (GMYC) model [32]. For bPTP, the maximum likelihood (ML) tree generated in RAxML was used as input in the bPTP webserver (https://species.h-its.org/ptp/), applying the default p-value model and standard settings. For GMYC, an ultrametric tree was first generated in BEAST v2.7 under a relaxed log-normal molecular clock with a Yule speciation prior. The single-threshold GMYC model was subsequently implemented in R using the “splits” package. By integrating both distance-based and phylogeny-based approaches, species boundaries were evaluated using complementary analytical frameworks that account for genetic divergence patterns and branching processes.

## Results

### Morphological identification of tick species

**Fig. 2:**
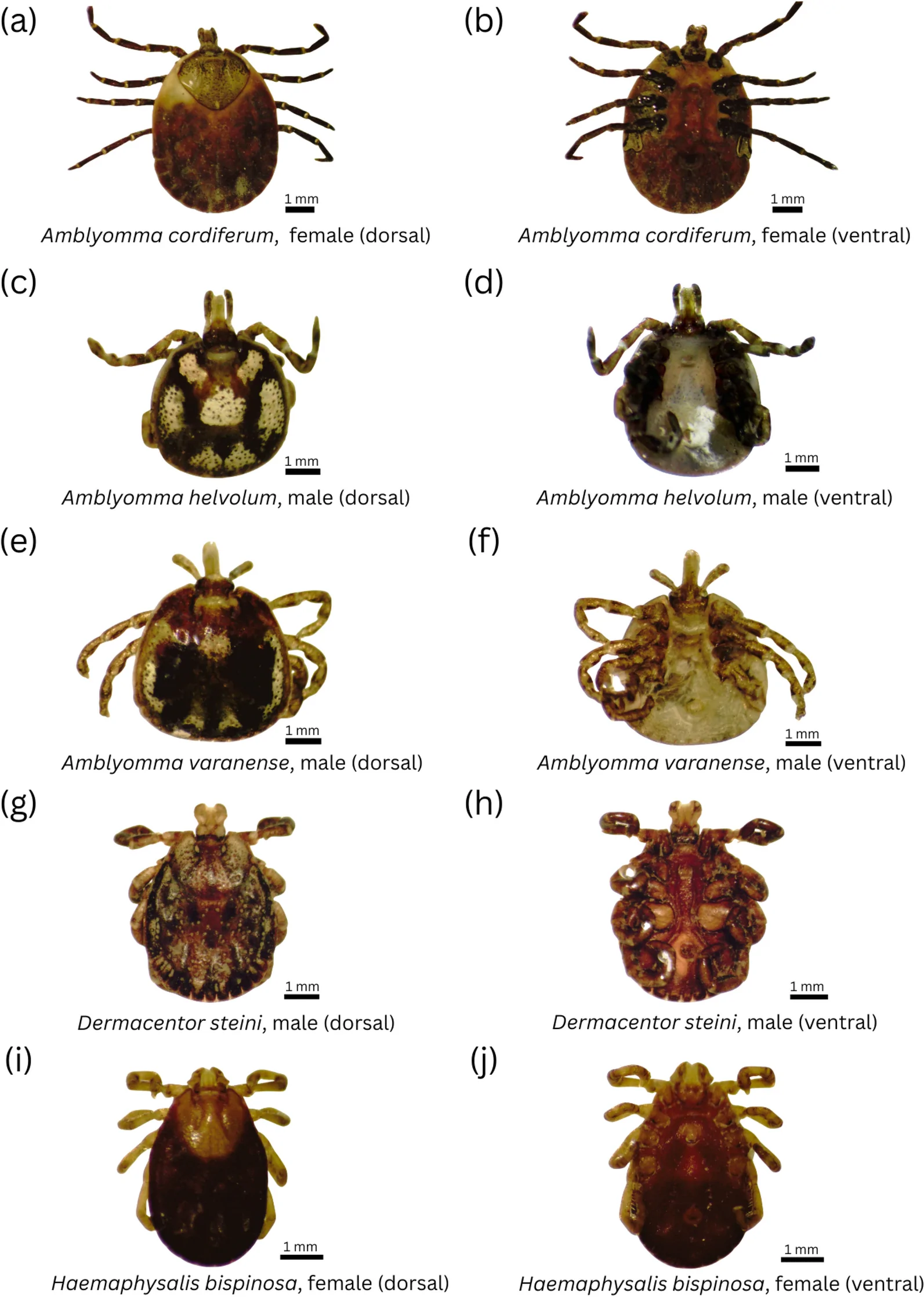
Representative morphological images of selected ixodid tick species identified in Peninsular Malaysia. Dorsal and ventral views are shown for: **a**, **b** *Amblyomma cordiferum* female; **c**, **d** *Amblyomma helvolum* male; **e**, **f** *Amblyomma varanense* male; **g**, **h** *Dermacentor steini* male; and **i**, **j** *Haemaphysalis bispinosa* female. Scale bars = 1 mm

**Fig. 3:**
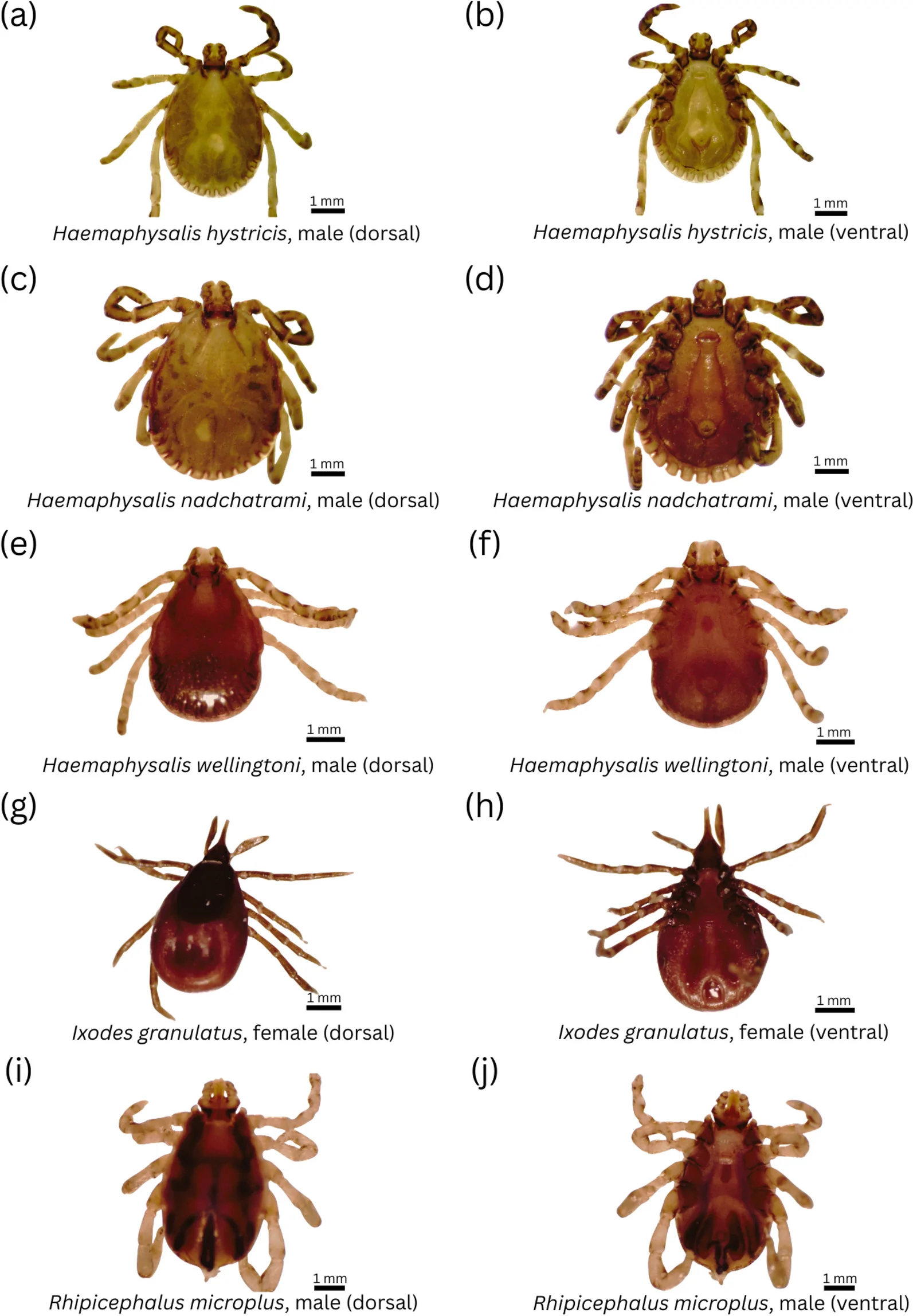
Representative morphological images of selected ixodid tick species identified in Peninsular Malaysia. Dorsal and ventral views are shown for: **a**, **b** *Haemaphysalis hystricis* male; **c**, **d** *Haemaphysalis nadchatrami* male; **e**, **f** *Haemaphysalis wellingtoni* male; **g**, **h** *Ixodes granulatus* female; and **i**, **j** *Rhipicephalus microplus* male. Scale bars = 1 mm

Morphological examination of all specimens identified 14 ixodid tick species representing five genera: *Amblyomma*, *Dermacentor*, *Haemaphysalis*, *Ixodes*, and *Rhipicephalus* (Table 3). Representative morphological plates illustrating selected species are presented in Figs. 2, 3. Diagnostic characters, including scutal ornamentation, palpal proportions, coxal spur configuration, and the presence or absence of eyes and festoons, were examined and used to assign specimens to their respective taxa following standard taxonomic keys.

**Table 3:** Number of morphologically identified species and operational taxonomic units (OTUs) inferred using two species delimitation methods, ASAP, ABGD, bPTP and GMYC across five tick genera from Peninsular Malaysia

Genus	Morphologically Identified Species	COI + 16S rDNA				COI				16S rDNA			
		ASAP	ABGD	bPTP	GMYC	ASAP	ABGD	bPTP	GMYC	ASAP	ABGD	bPTP	GMYC
*Amblyomma*	*A*. *cordiferum*	1	1	1	1	1	1	1	1	1	1	1	1
	*A*. *helvolum*	1	1	1	1	1	1	1	1	1	1	2	2
	*A*. *varanense*	1	1	3	4	1	1	1	1	1	1	4	4
*Dermacentor*	*D*.* auratus*	1	1	1	1	1	1	1	1	1	1	1	1
	*D. steini*	1	1	3	3	1	1	1	1	1	1	1	2
*Haemaphysalis*	*H*. *bispinosa*	1	1	1	1	1	1	1	1	1	1	1	1
	*H*. *hystricis*	1	1	1	2	1	1	1	1	1	1	1	1
	*H*. *nadchatrami*			1	1			1	1			1	1
	*H*. *semermis*	1	1	1	1	1	1	1	1	1	1	1	1
	*H*. *shimoga*	1	1	1	2	1	1	1	1	1	1	1	1
	*Haemaphysalis* sp	1	1	1	1	1	1	2	2	1	1	1	1
	*H*. *wellingtoni*	1	1	2	2	2	1	2	2	1	1	1	1
*Ixodes*	*I*. *granulatus*	1	1	3	4	2	1	2	3	1	1	3	3
*Rhipicephalus*	*R*. *microplus*	1	1	1	1	1	1	2	2	1	1	1	1
Total		13	13	21	25	15	13	18	19	13	13	20	21

OTUs inferred by ASAP and bPTP do not represent formal species and are used here for comparative analytical purposes only

### Amplification and sequencing of barcoding regions

A total of 319 specimens were successfully amplified and sequenced for both COI and 16S rDNA markers. The COI fragment (660 bp) contained 395 conserved and 167 variable sites, of which 10 were singleton and 157 were parsimony-informative. The 16S rDNA fragment (460 bp) comprised 409 conserved and 151 variable sites, including seven singleton and 144 parsimony-informative positions. The concatenated dataset of 1,120 bp (660 bp COI + 460 bp 16S rDNA) contained 806 conserved and 319 variable sites (17 singleton and 302 parsimony-informative) and was used for phylogenetic and barcode gap analyses.

### Phylogenetic analysis and species delimitation

**Fig. 4:**
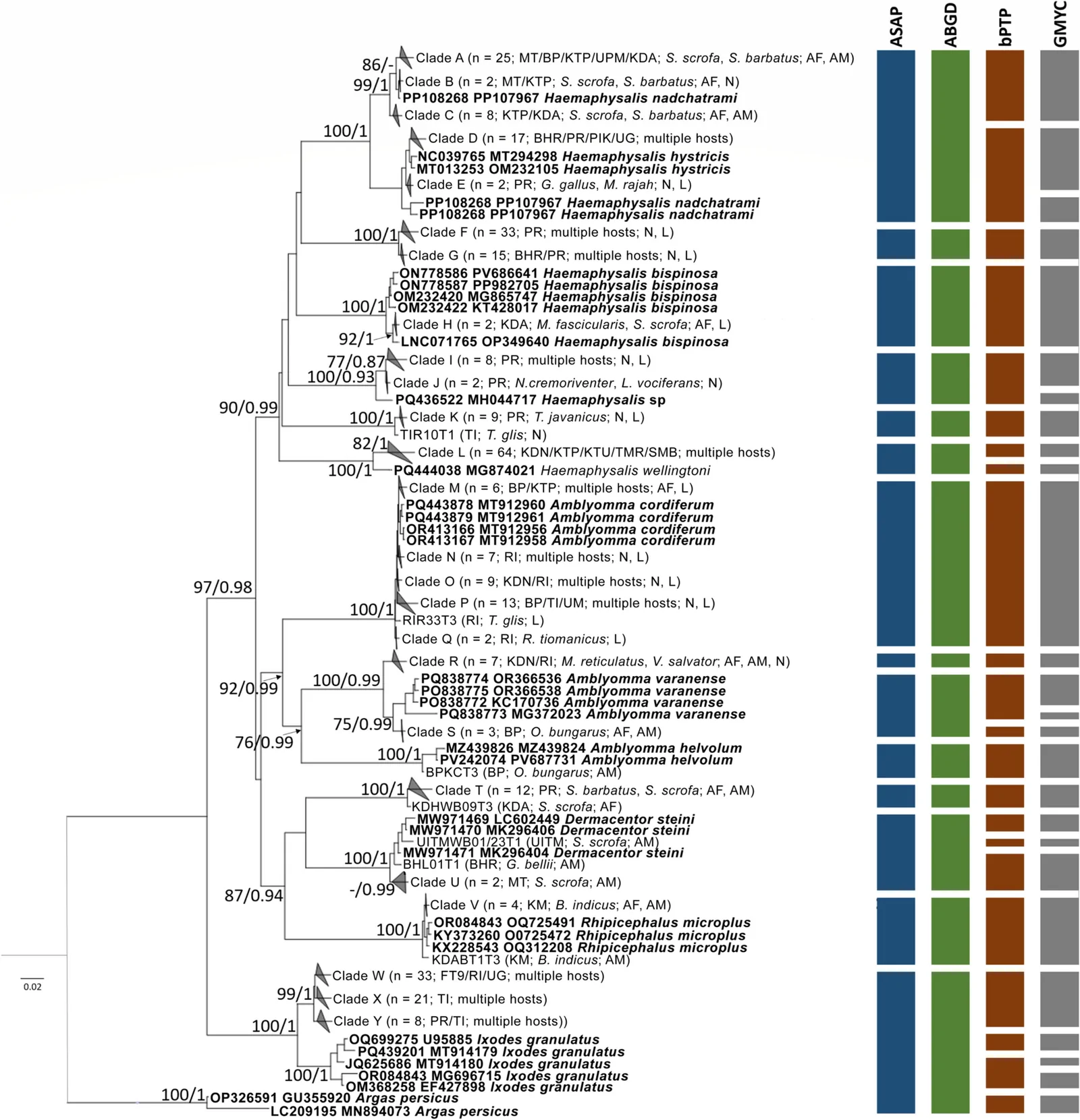
Simplified maximum likelihood phylogenetic tree showing representative sequences of hard ticks (Ixodidae) from Peninsular Malaysia based on concatenated COI + 16S rDNA sequences (1,120 bp). Collapsed clades (A–Y) represent groups of closely related sequences, with numbers indicating the total number of samples within each clade. Clade labels summarize sampling localities and host associations. Life stages are represented across clades unless otherwise specified and are abbreviated as follows: *L* larva, *N* nymph, *AF* adult female, *AM* adult male. Reference sequences obtained from GenBank are shown as separate tips and cluster within-specific clades (D, F, I, L, M, P, R, T, and V). Sequences from GenBank are shown in bold, while sequences generated in this study are shown in regular font. Numbers at nodes indicate maximum likelihood bootstrap support and Bayesian posterior probabilities. Species delimitation results from ASAP, ABGD, bPTP, and GMYC analyses are indicated by vertical bars. Full phylogenetic trees with all sequences are provided in Supplementary Figures S1–S10

Maximum likelihood phylogenetic analysis of the concatenated COI + 16S rDNA dataset revealed distinct and well-supported clades corresponding to the 14 morphologically identified species across five genera (Fig. 4). Newly generated sequences clustered with available GenBank references, and most species formed monophyletic groups with high bootstrap support (≥ 90%). Full phylogenetic trees based on all sequences are provided in Supplementary Figures S1–S10.

Across taxa, species delimitation results were generally congruent with morphological identification when using distance-based methods (ASAP and ABGD), whereas tree-based approaches (bPTP and GMYC) frequently inferred additional operational taxonomic units (OTUs), reflecting finer-scale genetic structuring (Table 3).

Within *Haemaphysalis*, most species were consistently recovered as single OTUs across methods, including *H. bispinosa*, *H. semermis*, and *H. nadchatrami*. In contrast, *H. hystricis*, *H. shimoga*, *Haemaphysalis* sp., and *H. wellingtoni* exhibited varying degrees of internal subdivision under tree-based methods, particularly GMYC and bPTP, while remaining largely resolved as single units under ASAP and ABGD.

Within *Amblyomma*, *A. cordiferum* and *A. helvolum* were consistently recovered as single OTU across most datasets. In contrast, *A. varanense* showed pronounced genetic structuring, with multiple OTUs inferred under bPTP and GMYC, particularly in the concatenated and 16S rDNA datasets, whereas distance-based methods generally supported a single species-level unit.

Within *Dermacentor*, *D. auratus* was consistently resolved as a single OTU across all methods, while *D. steini* exhibited internal subdivision under tree-based approaches in the concatenated and 16S rDNA datasets, but was recovered as a single unit under distance-based methods.

For *I. granulatus*, all sequences formed a strongly supported monophyletic clade; however, substantial intraspecific structuring was detected, with multiple OTUs inferred under bPTP and GMYC, in contrast to a single OTU recovered by ASAP and ABGD.

Similarly, *R. microplus* formed a well-supported monophyletic clade and was consistently recovered as a single OTU under concatenated and 16S rDNA analyses, although COI-based tree-based methods inferred limited subdivision.

Overall, species delimitation analyses showed clear method- and marker-dependent variation (Table 3). For the concatenated dataset, ASAP and ABGD recovered 13 OTUs, largely consistent with morphologically defined species, whereas bPTP and GMYC inferred 21 and 25 OTUs, respectively. Similar patterns were observed for single-marker datasets, with tree-based methods generally identifying higher numbers of OTUs. These results indicate that tree-based approaches are more sensitive to intraspecific genetic structure, whereas distance-based methods provide more conservative estimates of species boundaries that are largely congruent with morphological identification.

### Barcoding gap assessment

**Fig. 5:**
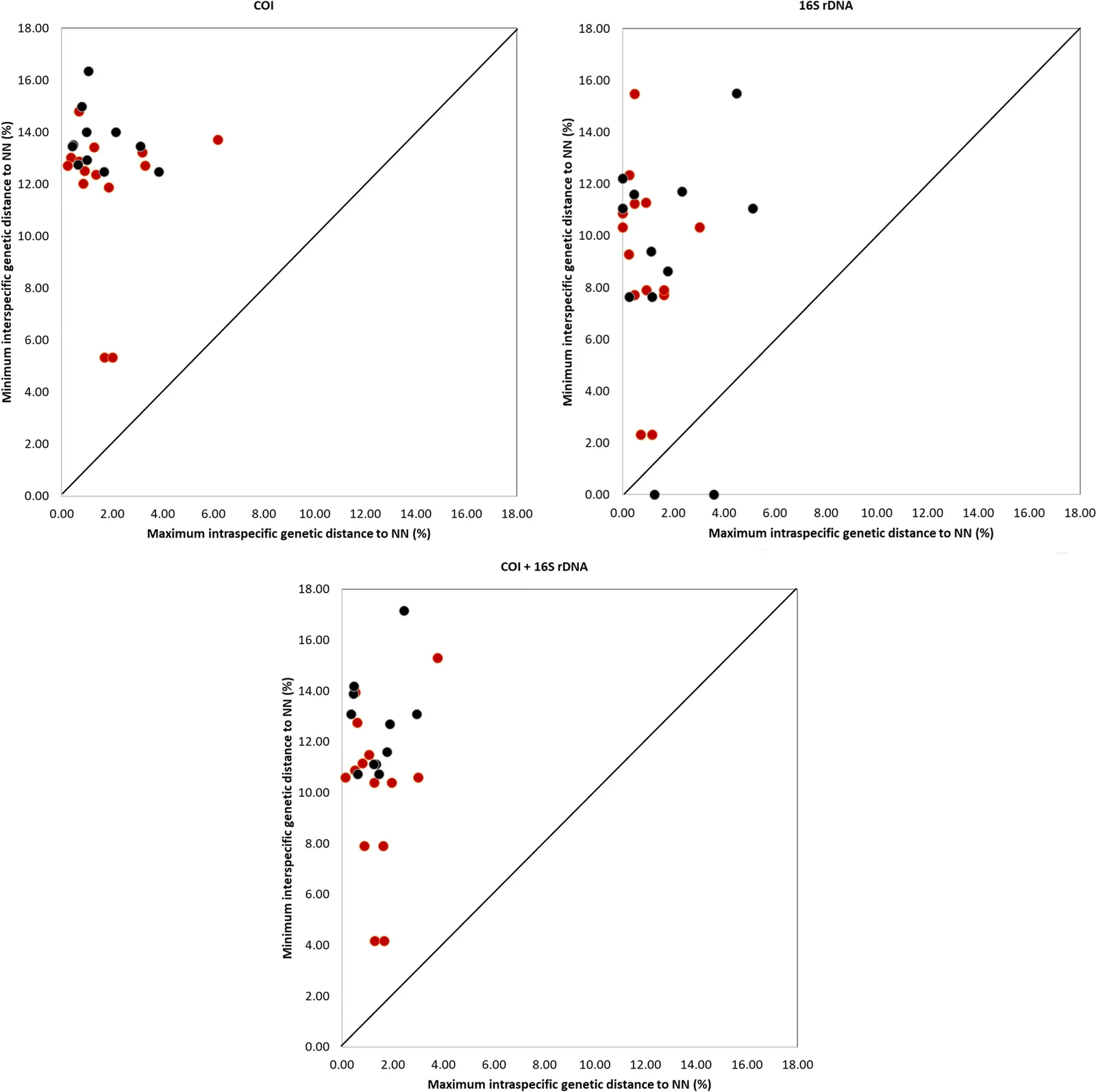
Scatter plots of maximum intraspecific versus minimum interspecific (nearest-neighbor) genetic distances for 319 sequences from 14 tick species across three barcoding markers: 16S rDNA, COI, and concatenated COI + 16S rDNA. Each point represents a species with more than one sequence; single-sequence species were excluded from the analysis. The 1:1 diagonal line indicates equal intra- and interspecific divergence. Species plotted above the line exhibit a DNA barcode gap. Red dots represent taxa containing newly generated sequences from this study, while black dots correspond to taxa represented only by GenBank reference sequences

Comparative assessment of maximum intraspecific and minimum interspecific (nearest-neighbor) pairwise distances across three markers (16S rDNA, COI, and concatenated COI + 16S rDNA) revealed clear barcode gaps for most taxa (Fig. 5). A barcode gap was defined as maximum intraspecific divergence being lower than minimum interspecific divergence.

For 16S rDNA, almost 86% of all species exhibited a barcode gap. Only two species, *H. hystricis* and *H. nadchatrami*, lacked a barcode gap, as their maximum intraspecific distances (3.58% and 1.25%, respectively) exceeded the minimum interspecific distance (0.00%). The highest intraspecific divergence was observed in *A. varanense* (5.12%), while *A. helvolum* and *Haemaphysalis* sp. had no detectable intraspecific variation.

Barcode gap patterns were generally more distinct with COI, with all species showing a barcode gap. The largest intraspecific divergence was again found in *A. varanense* (3.30%) and *I. granulatus* (6.18%), but these values remained well below their respective nearest-neighbor divergences (≥ 12.71%).

The combined marker also showed high resolving power, with 100% of all species exhibiting barcode gaps. Notably, *H. hystricis* and *H. nadchatrami*, which lacked gaps in the 16S rDNA-only marker, showed improved separation here (Max intra = 1.28–1.66%; Min inter = 4.17%). As in the single-marker results, *A. varanense* and *I. granulatus* had relatively high intraspecific divergence (2.95% and 3.76%, respectively), though both were well below their respective interspecific distances.

### Genetic divergence and pairwise distances

**Fig. 6:**
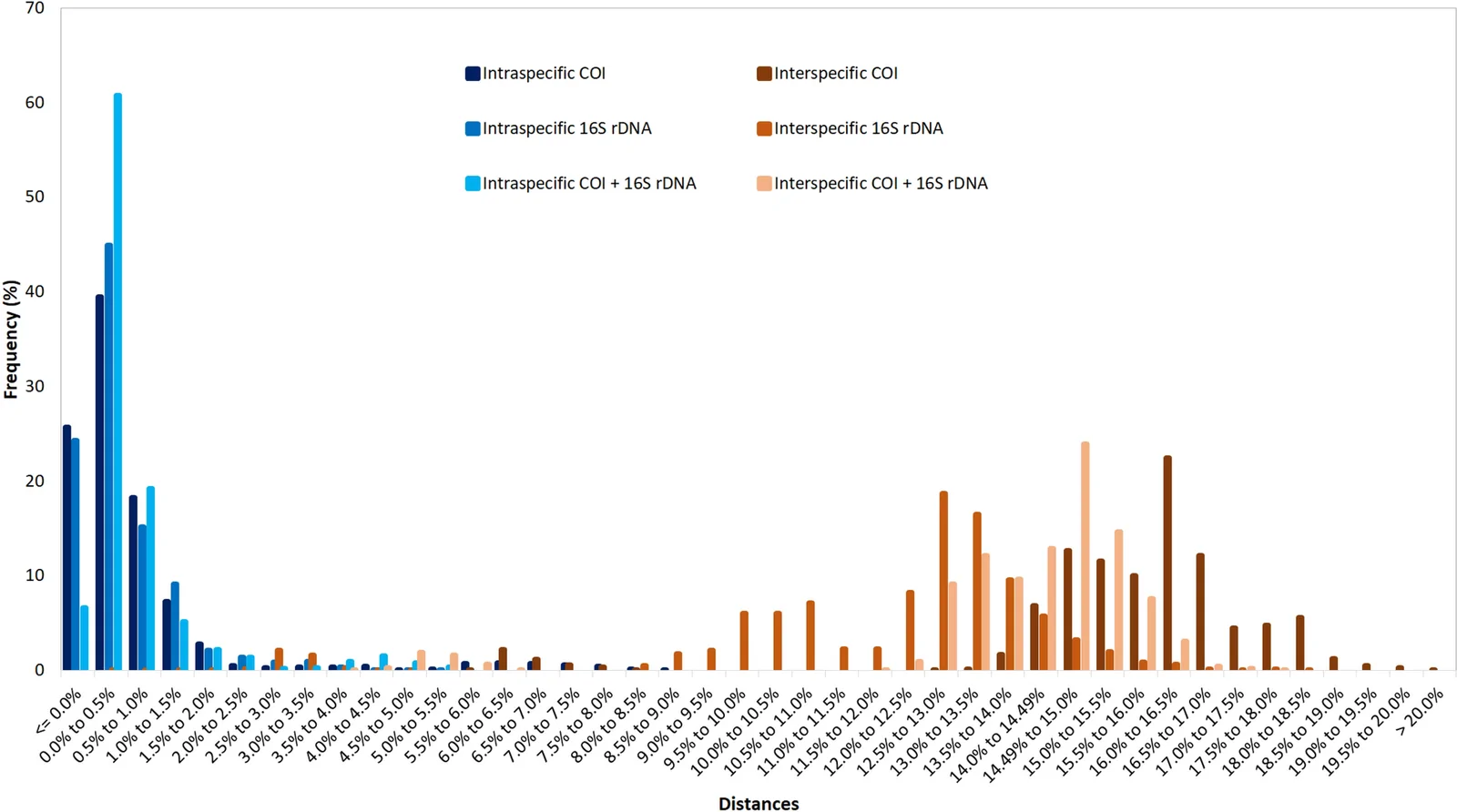
Distribution of pairwise intraspecific and interspecific genetic distances of 14 tick species across defined distance intervals for three barcoding markers: 16S rDNA, COI, and concatenated COI + 16S rDNA

The distribution of pairwise genetic distances across 14 tick species revealed distinct patterns between intraspecific and interspecific comparisons for the three barcoding markers (Fig. 6; Table 4). Intraspecific divergences were largely concentrated in lower genetic distance intervals. For 16S rDNA, 24.37% of comparisons showed 0% divergence, and 44.97% fell within the 0.0–0.5% range. Similarly, for COI, 25.74% of comparisons had 0% divergence, with 39.53% between 0.0 and 0.5%. The concatenated COI + 16S rDNA marker showed even stronger clustering at low divergence, with 60.76% of comparisons in the 0.0–0.5% range but fewer with exact matches (6.66% at 0%), reflecting greater sensitivity in capturing within-species variation.

**Table 4:** Mean and maximum intraspecific genetic divergences for 14 Malaysian tick species across three DNA barcoding markers: COI, 16S rDNA, and concatenated COI + 16S rDNA

Tick genus	Tick species	COI + 16S rDNA		COI		16S rDNA	
		*n*	Mean intraspecific divergence (maximum), %	*n*	Mean intraspecific divergence (maximum), %	*n*	Mean intraspecific divergence (maximum), %
*Amblyomma*	*A. cordiferum*	43†	0.14% (0.59%)	43†	0.03% (0.36%)	43†	0.29% (0.92%)
	*A. helvolum*	3†	0.12% (0.12%)	3†	0.23% (0.23%)	6†	0.00% (0.00%)
	***A. varanense***	15†	1.45% (3.01%)	15†	1.45% (3.30%)	15†	1.20% (3.02%)
*Dermacentor*	*D. auratus*	17†	0.46% (1.26%)	17†	0.63% (1.28%)	17†	0.25% (1.62%)
	***D. steini***	7†	0.96% (1.95%)	7†	1.78% (3.19%)	9†	0.19% (0.47%)
*Haemaphysalis*	*H. bispinosa*	7†	0.72% (1.06%)	7†	1.26% (1.86%)	7†	0.16% (0.24%)
	*H. hystricis*	24†	0.41% (1.28%)	24†	0.50% (1.70%)	24†	0.30% (0.70%)
	*H. nadchatrami*	36†	0.59% (1.66%)	36†	0.71% (2.01%)	40†	0.42% (1.16%)
	*H. shimoga*	15†	0.47% (1.62%)	15†	0.27% (0.68%)	15†	0.48% (1.62%)
	*H. semermis*	49†	0.27% (0.87%)	49†	0.33% (0.90%)	49†	0.20% (0.93%)
	*H. wellingtoni*	69†	0.11% (0.80%)	69†	0.16% (1.35%)	69†	0.03% (0.46%)
	*Haemaphysali* sp.	10	0.10% (0.49%)	11†	0.17% (0.85%)	10	0.00% (0.00%)
*Ixodes*	***I. granulatus***	67†	0.48% (3.76%)	67†	0.76% (6.18%)	67†	0.11% (0.46%)
*Rhipicephalus*	*R. microplus*	9†	0.17% (0.51%)	9†	0.22% (0.68%)	9†	0.09% (0.26%)
Overall		371		372		380	

*n* total number of sequences

^†^species with newly generated sequences and sequences retrieved from GenBank. Species highlighted in bold showed pronounced genetic structuring based on divergence values and tree-based species delimitation analyses

Interspecific distances were broader and shifted toward higher divergence ranges. For 16S rDNA, interspecific comparisons peaked between 12.5 and 13.5%, while COI distances ranged up to 22.5%, with the majority concentrated between 16.0 and 16.5%. The concatenated dataset also captured high interspecific divergence, peaking at 23.95% in the 14.49–15.0% range. Notably, some overlap between intra- and interspecific distances was observed, especially in the 16S rDNA dataset, where 2.16% of interspecific comparisons occurred within the 2.5–3.0% range. By contrast, the COI and COI + 16S rDNA datasets showed greater separation between intra- and interspecific divergence, indicating a clearer DNA barcode gap and stronger species discrimination.

Table 4 summarizes mean and maximum intraspecific divergences per species. *Ixodes granulatus* and *A. varanense* exhibited the highest maximum intraspecific divergences (6.18% and 3.30% for COI, respectively), while several species such as *A. helvolum*, *H. wellingtoni*, and *Haemaphysalis* sp. showed no detectable variation in 16S rDNA. Across all markers, mean intraspecific divergences remained below 1.5%, indicating overall genetic coherence within morphologically defined species.

### Species identification and assignment

The effectiveness of DNA barcoding in species identification was assessed using Best Match (BM), Best Close Match (BCM), and All Species Barcode (ASB) criteria for the three datasets (Table 5). The COI marker exhibited the highest performance, with 100.00% of sequences correctly identified under BM, 99.73% under BCM, and 99.73% under ASB. Only 0.27% returned no match under both BCM and ASB. No ambiguous assignments were recorded for COI.

**Table 5:** DNA barcode identifications of 14 Malaysian tick species using three DNA barcoding markers (COI, 16S rDNA, and concatenated COI + 16S rDNA) evaluated with TaxonDNA functions: Best Match (BM), Best Close Match (BCM), and All Species Barcode (ASB)

	Best match (%)			Best close match (%)				All species barcode (%)			
	Correct	Ambigous	Incorrect	Correct	Ambigous	Incorrect	No match	Correct	Ambigous	Incorrect	No match
COI (*n* = 371)	100.00	0.00	0.00	99.73	0.00	0.00	0.27	99.73	0.00	0.00	0.27
16S rDNA (*n* = 379)	99.21	0.26	0.53	99.12	0.26	0.62	0.00	83.15	16.59	0.26	0.00
COI + 16S rDNA (*n* = 370)	99.72	0.00	0.28	98.11	0.00	0.00	1.89	37.75	60.37	0.00	1.88

The 16S rDNA marker also yielded high identification success, with 99.21% correct identifications under BM and 99.12% under BCM. A small proportion of sequences were incorrectly assigned (0.53%) under BM and 0.62% under BCM, while 0.26% were recorded as ambiguous under both criteria. ASB results for 16S rDNA were comparatively lower, with 83.15% correct assignments, 16.59% ambiguous matches, and 0.26% incorrect identifications.

The concatenated COI + 16S rDNA dataset produced 99.72% correct identifications under BM and 98.11% under BCM. However, ASB performance was substantially lower, with only 37.75% correct assignments and a high proportion of ambiguous matches (60.37%). A small proportion of sequences returned no matches under BCM (1.89%) and ASB (1.88%). These results indicate that although concatenated markers maintained high accuracy under BM and BCM, they introduced greater ambiguity under the more stringent ASB criterion.

Among the three datasets, COI provided the highest species identification accuracy (100.00% under BM and 99.73% under BCM), followed by the concatenated COI + 16S rDNA dataset (99.72% and 98.11%, respectively) and 16S rDNA (99.21% and 99.12%, respectively). Although concatenation slightly improved BM accuracy, it substantially increased ambiguous matches under the ASB criterion (60.37%). This pattern indicates that concatenated multilocus datasets, while useful for phylogenetic reconstruction, may reduce assignment clarity under strict barcode identification criteria. In contrast, the single-locus COI marker provided the most consistent and reliable performance for routine DNA barcoding.

## Discussion

This study provides a comprehensive integrative taxonomic evaluation of hard ticks in Peninsular Malaysia. By combining morphological diagnostics with molecular phylogenetics, species delimitation analyses, and DNA barcode gap assessment, we evaluated 14 morphologically defined species from five genera (*Amblyomma*, *Dermacentor*, *Haemaphysalis*, *Ixodes*, and *Rhipicephalus*) using mitochondrial COI and 16S rDNA markers. Overall, the patterns observed in phylogenetic reconstruction, barcode gap analyses, and species delimitation approaches revealed strong concordance between morphological and molecular data, while also identifying pronounced mitochondrial genetic structuring within several taxa.

### Phylogenetic structure and species delimitation

Phylogenetic and species delimitation analyses revealed clear patterns of mitochondrial structuring across multiple tick genera, indicating varying levels of lineage divergence within morphologically defined species. Across datasets, distance-based methods (ASAP and ABGD) consistently recovered species boundaries congruent with morphological identification, whereas tree-based approaches (bPTP and GMYC) inferred higher numbers of operational taxonomic units, reflecting sensitivity to intraspecific mitochondrial variation [33].

Genetic structuring was most evident within the genera *Haemaphysalis*, *Amblyomma*, and *Dermacentor*. Within *Haemaphysalis*, several species, including *H. hystricis*, *H. bispinosa*, and *H. nadchatrami*, were subdivided into multiple OTUs under tree-based methods, whereas ASAP and ABGD largely recovered single entities. Marker-specific differences were also evident; for example, *H. bispinosa* was partitioned into two lineages based on COI but formed a single lineage using 16S rDNA, consistent with the higher variability of COI relative to 16S rDNA [7]. Similar mitochondrial structuring has been reported in several *Haemaphysalis* species and may reflect geographic or ecological differentiation within broadly distributed taxa.

Within *Amblyomma*, both *A. helvolum* and *A. varanense* exhibited pronounced mitochondrial structuring. *Amblyomma varanense* was subdivided into multiple lineages across datasets, particularly under tree-based approaches, likely reflecting geographic or host-associated differentiation within a single morphospecies. Comparable mitochondrial structuring has been reported in *A. testudinarium*, where strong genetic separation between regional populations was detected without formal taxonomic revision [34].

In *Dermacentor*, both *D. auratus* and *D. steini* were subdivided into multiple OTUs under tree-based analyses despite forming well-supported morphological clades. Similar patterns of mitochondrial lineage subdivision have been reported in other tick taxa and are often interpreted as evidence of population-level genetic structuring rather than definitive species boundaries [15, 35]. In the absence of corresponding morphological differentiation, the lineages detected here are more conservatively interpreted as intraspecific mitochondrial variation that warrants further investigation using nuclear markers.

Although *I. granulatus* was recovered as a well-supported monophyletic clade, multiple lineages were detected under tree-based delimitation. Previous studies have reported pronounced mitochondrial divergence between populations from different geographic regions, including Taiwan and Malaysia, suggesting restricted gene flow at broader spatial scales [36]. In the present study, however, the subdivisions detected within Peninsular Malaysia are more plausibly interpreted as population-level mitochondrial structuring rather than evidence of multiple species.

Collectively, these findings demonstrate that mitochondrial markers are effective for resolving species-level relationships but are also sensitive to intraspecific variation [37]. While tree-based methods highlight fine-scale genetic structuring, distance-based approaches provide more conservative estimates of species boundaries [33]. Integrating these complementary approaches allows for a more balanced interpretation of tick diversity, distinguishing population structure from species-level divergence.

### Congruence and discrepancies between morphological and molecular data

The phylogenetic and species delimitation patterns observed across taxa provide a framework for evaluating the level of agreement between morphological and molecular approaches. Morphological identification based on genus- and species-specific characters (e.g., palpal segmentation, coxal spurs, and scutal ornamentation) was largely corroborated by molecular clustering, with most taxa forming well-supported monophyletic clades. Species such as *A. cordiferum*, *R. microplus*, and *H. semermis* were consistently recovered as single entities across both distance-based (ASAP, ABGD) and tree-based (bPTP, GMYC) delimitation methods. These findings support the continued use of established morphological keys when complemented by molecular validation [13, 38].

However, discrepancies among delimitation methods were observed in several taxa. Tree-based approaches (bPTP and GMYC) frequently inferred a higher number of operational taxonomic units (OTUs) compared to distance-based methods (ASAP and ABGD). For example, *A. varanense*, *I. granulatus*, and *D. steini* were subdivided into multiple lineages under tree-based analyses, whereas ASAP and ABGD largely recovered these taxa as single species-level units. Despite this subdivision, mean intraspecific divergence values remained low (< 2%), although maximum COI divergence reached up to 3.30% in *A. varanense* and 3.19% in *D. steini*, suggesting moderate mitochondrial structuring rather than clear-cut species boundaries.

A similar pattern was observed in *H. wellingtoni*, where lineage subdivision was detected under tree-based methods despite low mean divergence values, while ASAP and ABGD supported a single entity. In contrast, *H. hystricis* and *H. nadchatrami* showed limited genetic divergence and were consistently resolved as single units by distance-based approaches. These patterns indicate that tree-based splits likely reflect population-level subdivision rather than distinct species.

The higher OTU counts recovered by bPTP and GMYC likely reflect the sensitivity of coalescent-based models to intraspecific genetic structure, uneven sampling, and mitochondrial demographic history. The GMYC method, in particular, is known to over-split lineages when branch length variation reflects population processes rather than speciation events [33], while bPTP may partition geographically structured haplotypes into separate entities when substitution rates vary among branches. In contrast, distance-based methods such as ASAP and ABGD rely on barcode gap detection and are generally more conservative, explaining their closer agreement with morphological species boundaries.

Mitochondrial structuring of this nature has increasingly been reported in ticks and may arise from ecological divergence, host associations, restricted dispersal, or geographic isolation [35]. Comparable findings were reported in *R. microplus* from Peninsular Malaysia, where mitochondrial data revealed genetically distinct assemblages within morphologically similar ticks, suggesting the presence of cryptic diversity [15]. In the present study, however, the observed mitochondrial structuring is interpreted more conservatively as population-level differentiation because the overall genetic divergence remained relatively low and morphological differentiation was not evident.

Overall, although multiple OTUs were inferred within several morphologically defined taxa, the combined evidence from genetic distances, barcode gap analyses, and congruence with morphology does not support taxonomic revision. The inferred OTUs are therefore interpreted conservatively as hypotheses of mitochondrial lineage structuring that warrant further investigation using nuclear markers and integrative taxonomic approaches. These findings underscore the importance of interpreting species delimitation results within a multimethod and multievidence framework, particularly in ixodid ticks where mitochondrial population structuring can be pronounced.

### Barcoding gap and genetic divergence

The barcoding gap analysis reinforced the discriminatory power of the COI and concatenated COI + 16S rDNA markers for distinguishing morphologically defined species. A clear separation between intra- and interspecific genetic distances was observed in 100% of species using COI and COI + 16S rDNA, while 16S rDNA alone failed to discriminate between *H. hystricis* and *H. nadchatrami*, due to overlapping distances. These findings confirm that while 16S rDNA is informative for genus-level placement, it lacks the variability necessary for resolving closely related species, consistent with previous tick barcoding studies [8, 39].

Although most taxa showed low intraspecific divergence (< 1.5%), several species exceeded the typical 2–3% threshold used for species delimitation. Notably, *I. granulatus* reached 6.18% and *A. varanense* 3.30% for COI, indicating substantial intraspecific mitochondrial divergence. These high divergences, when coupled with multiple bPTP-inferred lineages, may reflect historical isolation, restricted gene flow, or structured population dynamics. In contrast, species such as *H. semermis*, *Haemaphysalis* sp., and *A. helvolum* exhibited no detectable divergence in 16S rDNA, possibly due to recent divergence or slower evolutionary rates in this marker. The COI and concatenated datasets showed clearer separation between intra- and interspecific distances, with most interspecific values exceeding 12%, supporting the use of COI as a reliable DNA barcode for tick species [8, 40].

### Species identification accuracy

TaxonDNA analysis confirmed that COI is the most accurate marker for species identification. It achieved 100% accuracy under the Best Match criterion and more than 99% under Best Close Match and All Species Barcode criteria. In contrast, 16S rDNA had lower identification success, with up to 16.59% ambiguous matches under the ASB test. The concatenated marker performed well in phylogenetic and barcode gap analyses but yielded reduced accuracy (37.75%) in ASB. This reduction likely reflects the increased influence of within-species variation across longer concatenated sequences under the stringent ASB criterion.

These results support COI as the most effective DNA barcode for tick identification, particularly when used with validated morphological keys and high-quality reference libraries [8, 41]. While concatenated markers enhance phylogenetic inference, COI remains the optimal marker for routine species assignment.

### Taxonomic implications and future directions

The detection of genetically structured lineages within several tick species highlights the complexity of tick population genetics in Southeast Asia but should not be conflated with species boundaries in the absence of integrative evidence. Mitochondrial markers are particularly sensitive to historical isolation, host-associated structuring, and demographic processes, which can generate pronounced intraspecific divergence without corresponding speciation [37, 42]. Accordingly, the OTUs identified in this study are best regarded as candidate genetic lineages that provide a framework for hypothesis-driven investigation, rather than as evidence of distinct species.

Despite this taxonomic caution, the presence of genetically divergent lineages within medically and veterinary important genera has meaningful implications for biodiversity assessment and vector surveillance. Lineages that are morphologically indistinguishable may nonetheless differ in ecological traits, host associations, or vector competence, which could influence pathogen transmission dynamics [43]. From a public health perspective, unrecognized genetic structuring within vector populations may therefore obscure heterogeneity in disease risk, even when formal species boundaries remain unchanged.

In particular, structured mitochondrial variation observed within the genera *Haemaphysalis* and *Dermacentor* warrants further investigation, as both genera include established vectors of zoonotic pathogens such as *Rickettsia*, *Babesia*, and *Theileria*. If genetically differentiated lineages within these taxa exhibit variation in host preference or pathogen susceptibility, this could have direct implications for understanding local disease ecology and refining surveillance strategies in regions where tick identification relies primarily on morphology.

Overall, these findings underscore the need for expanded geographic and host sampling, coupled with multilocus nuclear markers or genomic approaches, to determine whether the observed mitochondrial lineages represent stable evolutionary units, ecotypes, or structured populations within broadly distributed tick species. Integrative frameworks that combine morphology, multilocus genetics, and ecological data will be essential for resolving tick systematics and for improving vector surveillance and tick-borne disease risk assessment in Southeast Asia.

## Conclusions

In conclusion, this integrative taxonomic study confirms the overall reliability of morphology-based identification of ixodid ticks in Peninsular Malaysia while demonstrating the added value of mitochondrial DNA barcoding for detecting genetic structure within species. Although species delimitation analyses revealed multiple OTUs in several taxa, these units are interpreted conservatively as hypotheses of intraspecific genetic structuring rather than evidence of undiscovered species. No formal taxonomic changes are proposed based on the present data. While COI remains the most effective marker for routine species identification, concatenated mitochondrial datasets improve phylogenetic inference but may increase ambiguity under strict barcoding criteria. Together, these findings emphasize the need for integrative approaches that combine morphology, multilocus genetics, and ecological data to accurately resolve species boundaries and to support tick biodiversity assessments, risk-based surveillance, and evidence-based vector control strategies in Southeast Asia.

## Supplementary Information

Supplementary material 1: Fig. S1 Maximum likelihood (ML) subtree of *Haemaphysalis nadchatrami* based on concatenated COI + 16S rDNA sequences (1,120 bp). Numbers at nodes indicate ML bootstrap support and Bayesian posterior probabilities. Sequences from GenBank are shown in bold, while sequences generated in this study are in regular font. Vertical bars indicate species delimitation results from ASAP, ABGD, bPTP, and GMYC.

Supplementary material 2: Fig. S2 Maximum likelihood (ML) subtree of *Haemaphysalis hystricis* based on concatenated COI + 16S rDNA sequences (1,120 bp). Numbers at nodes indicate ML bootstrap support and Bayesian posterior probabilities. Sequences from GenBank are shown in bold, while sequences generated in this study are in regular font. Vertical bars indicate species delimitation results from ASAP, ABGD, bPTP, and GMYC.

Supplementary material 3: Fig. S3 Maximum likelihood (ML) subtree of *Haemaphysalis semermis* based on concatenated COI + 16S rDNA sequences (1,120 bp). Numbers at nodes indicate ML bootstrap support and Bayesian posterior probabilities. Sequences from GenBank are shown in bold, while sequences generated in this study are in regular font. Vertical bars indicate species delimitation results from ASAP, ABGD, bPTP, and GMYC.

Supplementary material 4: Fig. S4 Maximum likelihood (ML) subtree of *Haemaphysalis bispinosa*, *H. shimoga*, and *Haemaphysalis* sp. based on concatenated COI + 16S rDNA sequences (1,120 bp). Numbers at nodes indicate ML bootstrap support and Bayesian posterior probabilities. Sequences from GenBank are shown in bold, while sequences generated in this study are in regular font. Vertical bars indicate species delimitation results from ASAP, ABGD, bPTP, and GMYC.

Supplementary material 5: Fig. S5 Maximum likelihood (ML) subtree of *Haemaphysalis wellingtoni* based on concatenated COI + 16S rDNA sequences (1,120 bp). Numbers at nodes indicate ML bootstrap support and Bayesian posterior probabilities. Sequences from GenBank are shown in bold, while sequences generated in this study are in regular font. Vertical bars indicate species delimitation results from ASAP, ABGD, bPTP, and GMYC.

Supplementary material 6: Fig. S6 Maximum likelihood (ML) subtree of *Haemaphysalis wellingtoni* (continued). Numbers at nodes indicate ML bootstrap support and Bayesian posterior probabilities. Sequences from GenBank are shown in bold, while sequences generated in this study are in regular font. Vertical bars indicate species delimitation results from ASAP, ABGD, bPTP, and GMYC.

Supplementary material 7: Fig. S7 Maximum likelihood (ML) subtree of *Amblyomma cordiferum* based on concatenated COI + 16S rDNA sequences (1,120 bp). Numbers at nodes indicate ML bootstrap support and Bayesian posterior probabilities. Sequences from GenBank are shown in bold, while sequences generated in this study are in regular font. Vertical bars indicate species delimitation results from ASAP, ABGD, bPTP, and GMYC.

Supplementary material 8: Fig. S8 Maximum likelihood (ML) subtree of *Amblyomma varanense*, *A. helvolum*, *Dermacentor auratus*, and *D. steini* based on concatenated COI + 16S rDNA sequences (1,120 bp). Numbers at nodes indicate ML bootstrap support and Bayesian posterior probabilities. Sequences from GenBank are shown in bold, while sequences generated in this study are in regular font. Vertical bars indicate species delimitation results from ASAP, ABGD, bPTP, and GMYC.

Supplementary material 9: Fig. S9 Maximum likelihood (ML) subtree of *Rhipicephalus microplus* and *Ixodes granulatus* based on concatenated COI + 16S rDNA sequences (1,120 bp). Numbers at nodes indicate ML bootstrap support and Bayesian posterior probabilities. Sequences from GenBank are shown in bold, while sequences generated in this study are in regular font. Vertical bars indicate species delimitation results from ASAP, ABGD, bPTP, and GMYC.

Supplementary material 10: Fig. S10 Maximum likelihood (ML) subtree of *Ixodes granulatus* (continued) and *Argas persicus* (outgroup) based on concatenated COI + 16S rDNA sequences (1,120 bp). Numbers at nodes indicate ML bootstrap support and Bayesian posterior probabilities. Sequences from GenBank are shown in bold, while sequences generated in this study are in regular font. Vertical bars indicate species delimitation results from ASAP, ABGD, bPTP, and GMYC.

## Data Availability

Data supporting the main conclusions of this study are included in the manuscript.

## Abbreviations

COI: Cytochrome c oxidase I
16S rDNA: 16S ribosomal DNA
ASAP: Assemble Species by Automatic Partitioning
ABGD: Automatic Barcode Gap Discovery
bPTP: Bayesian Poisson tree processes
GMYC: General Mixed Yule-Coalescent
OTUs: Operational taxonomic units
IACUC: Institutional Animal Care and Use Committee
MEGA: Molecular evolutionary genetics analysis
BIC: Bayesian information criterion
TN93 + G: Tamura–Nei model with gamma-distributed
ML: Maximum Likelihood
CIPRES: Cyber Infrastructure for Phylogenetic Research Science Gateway
BI: Bayesian inference
BEAST: Bayesian evolutionary analysis by sampling trees
HKY: Hasegawa–Kishono–Yano
MCMC: Markov Chain Monte Carlo
MCC: Maximum clade credibility
iTOL: Interactive tree of life
K2P: Kimura 2-parameter
NN: Nearest neighbor
BM: Best match
BCM: Best close match
ASB: All species barcode
Af: Adult female
Am: Adult male
N: Nymph
L: Larva
